# Behavioral-metabolic phenotypes and differential risks of diabetic kidney disease and peripheral neuropathy in type 2 diabetes: a latent class analysis

**DOI:** 10.3389/fendo.2026.1962966

**Published:** 2026-10-15

**Authors:** Yue Wang, Zijian Liu, Zhengshan Zhang, An Hu, Lin Liu, Shuanghui Zeng, Ping Liu, Yahui Wang, Luying Sun

**Affiliations:** 1 Beijing University of Chinese Medicine, Beijing, China; 2 Department of Nephrology and Endocrinology, Dongzhimen Hospital, Beijing University of Chinese Medicine, Beijing, China; 3 Integrated Chinese and Western Medicine Metabolic Disease Research Center, Fangshan District Traditional Chinese Medicine Hospital, Beijing, China

**Keywords:** behavioral-metabolic phenotype, diabetic kidney disease, diabetic peripheral neuropathy, latent class analysis, socioeconomic status, type 2 diabetes mellitus

## Abstract

**Background:**

Behavioral and metabolic risk factors frequently coexist in type 2 diabetes mellitus (T2DM), but their combined patterns and associations with microvascular complications remain insufficiently characterized.

**Methods:**

This cohort study included 706 individuals with T2DM. Outcome-specific risk sets were established by excluding participants with prevalent diabetic kidney disease (DKD) or diabetic peripheral neuropathy (DPN) at baseline. Behavioral and metabolic factors were classified using latent class analysis. Multinomial logistic regression and Cox models, alongside socioeconomic status (SES)-stratified analyses, assessed associations among phenotypes, SES, and incident complications.

**Results:**

Three behavioral-metabolic phenotypes were identified: the Behavioral-Visceral Adiposity (BVA) phenotype (14.3%), characterized by unhealthy lifestyle behaviors, lower BMI, and higher visceral adiposity; the Obesity-Related Metabolic Risk (OMR) phenotype (39.8%), characterized by obesity, physical inactivity, insulin resistance, and suboptimal blood pressure control; and the Low-Risk (LR) phenotype (45.9%). Compared with the LR phenotype, the OMR phenotype showed increased risks of incident DKD (HR = 2.01; 95% CI = 1.32–3.04) and DPN (HR = 2.06; 95% CI = 1.18–3.58). The BVA phenotype was associated with increased risk of DPN (HR = 2.47; 95% CI = 1.19–5.12). SES was independently associated with phenotype membership. Associations between the OMR phenotype and DKD or DPN were mainly observed among individuals with low SES, whereas the association between the BVA phenotype and DPN was mainly observed among individuals with high SES.

**Conclusions:**

Three distinct behavioral-metabolic phenotypes were identified among individuals with T2DM and showed differential associations with DKD and DPN risks. Integrating behavioral patterns, metabolic profiles, and socioeconomic context into risk stratification strategies may enhance early identification and individualized prevention of microvascular complications.

## 1 Introduction

Type 2 diabetes mellitus (T2DM) represents a major global public health challenge, with rapidly increasing prevalence and substantial impacts on healthcare systems and society. According to the International Diabetes Federation (IDF), approximately 537 million adults were living with diabetes worldwide in 2021, and this number is projected to increase to nearly 700 million by 2045, with T2DM accounting for more than 90% of all diabetes cases (1). China has one of the largest populations of individuals living with diabetes worldwide. The continued increase in diabetes prevalence has imposed substantial burdens on healthcare systems and socioeconomic development (2). Beyond hyperglycemia itself, diabetic microvascular complications, including diabetic kidney disease (DKD) and diabetic peripheral neuropathy (DPN), are major contributors to impaired quality of life, disability, and premature mortality among individuals with diabetes (3–5). Given that established DKD and DPN are often difficult to reverse, early identification of individuals at high risk and implementation of targeted interventions are essential to delay disease progression (6).

Previous studies have demonstrated that the development of diabetic microvascular complications is influenced not only by glycemic control but also by the cumulative effects of multiple behavioral and metabolic risk factors (7, 8). From a behavioral perspective, unhealthy lifestyle factors, including smoking, excessive alcohol consumption, physical inactivity, high sodium intake, and insufficient fruit and vegetable consumption, may contribute to microvascular damage through mechanisms involving oxidative stress, inflammation, and endothelial dysfunction (9–11). From a metabolic perspective, abnormalities such as obesity, insulin resistance, hypertension, and dyslipidemia may further accelerate the development of diabetic microvascular complications through pathways involving lipotoxicity, chronic low-grade inflammation, and glomerular hyperfiltration (12–14). Importantly, these risk factors rarely occur in isolation; instead, they frequently coexist within individuals, forming complex behavioral-metabolic risk patterns. However, most previous studies have focused on individual risk factors or simple composite scores (11, 13), which may not adequately capture the heterogeneity resulting from the coexistence of multiple behavioral and metabolic characteristics among individuals with T2DM.

Latent class analysis (LCA) is a probability-based, unsupervised statistical approach that identifies unobserved subgroups within a population based on multiple observed indicators, enabling the characterization of distinct risk-factor clustering patterns and population heterogeneity (15). Compared with conventional approaches that focus on individual risk factors, LCA allows simultaneous consideration of coexisting behavioral and metabolic characteristics and provides a comprehensive framework for identifying subgroups with distinct risk profiles (16). However, few studies have applied LCA to integrate behavioral and metabolic data for identifying risk phenotypes among individuals with T2DM and evaluating their associations with incident DKD and DPN.

Beyond behavioral and metabolic determinants, socioeconomic status (SES) has been widely recognized as an important social determinant of health influencing diabetes development, progression, and outcomes (17). Individuals with lower SES often experience poorer dietary quality, limited access to healthcare resources, lower health literacy, and greater difficulties in maintaining healthy lifestyles, which may increase their susceptibility to chronic diabetic complications (17). Importantly, SES may function not only as an independent determinant of diabetic complications but also as a factor influencing the clustering patterns of behavioral and metabolic risk factors.

Therefore, this study aimed to: (1) identify behavioral-metabolic phenotypes among individuals with T2DM using LCA; (2) evaluate the associations between these phenotypes and the risks of incident DKD and DPN; and (3) analyze the influence of SES on the assignment of behavioral-metabolic phenotypes, and further explore the association between behavioral-metabolic phenotypes and the risk of diabetic microvascular complications across different SES levels. This study may provide insights into precision risk stratification and individualized prevention strategies for microvascular complications among individuals with T2DM.

## 2 Materials and methods

### 2.1 Study design and participants

This retrospective cohort study included individuals with T2DM who were recruited from the National Metabolic Management Center (MMC) at Fangshan Hospital, Beijing University of Chinese Medicine, between January 2021 and December 2025. Eligible participants were required to meet the diagnostic criteria for T2DM according to the ADA 2026 guidelines and have comprehensive baseline behavioral and metabolic data for latent class analysis (18). To evaluate incident complications, we subsequently excluded participants with pre-existing DKD at baseline when establishing the DKD risk set, and excluded those with pre-existing DPN at baseline when establishing the DPN risk set. Finally, a total of 706 participants were included in the baseline phenotypic clustering. Participant selection and derivation of the analytic cohorts are shown in **Figure 1**. The study protocol was approved by the Institutional Review Board of Fangshan Hospital, Beijing University of Chinese Medicine (approval number: FZYLK-2026-019).

**Figure 1:**
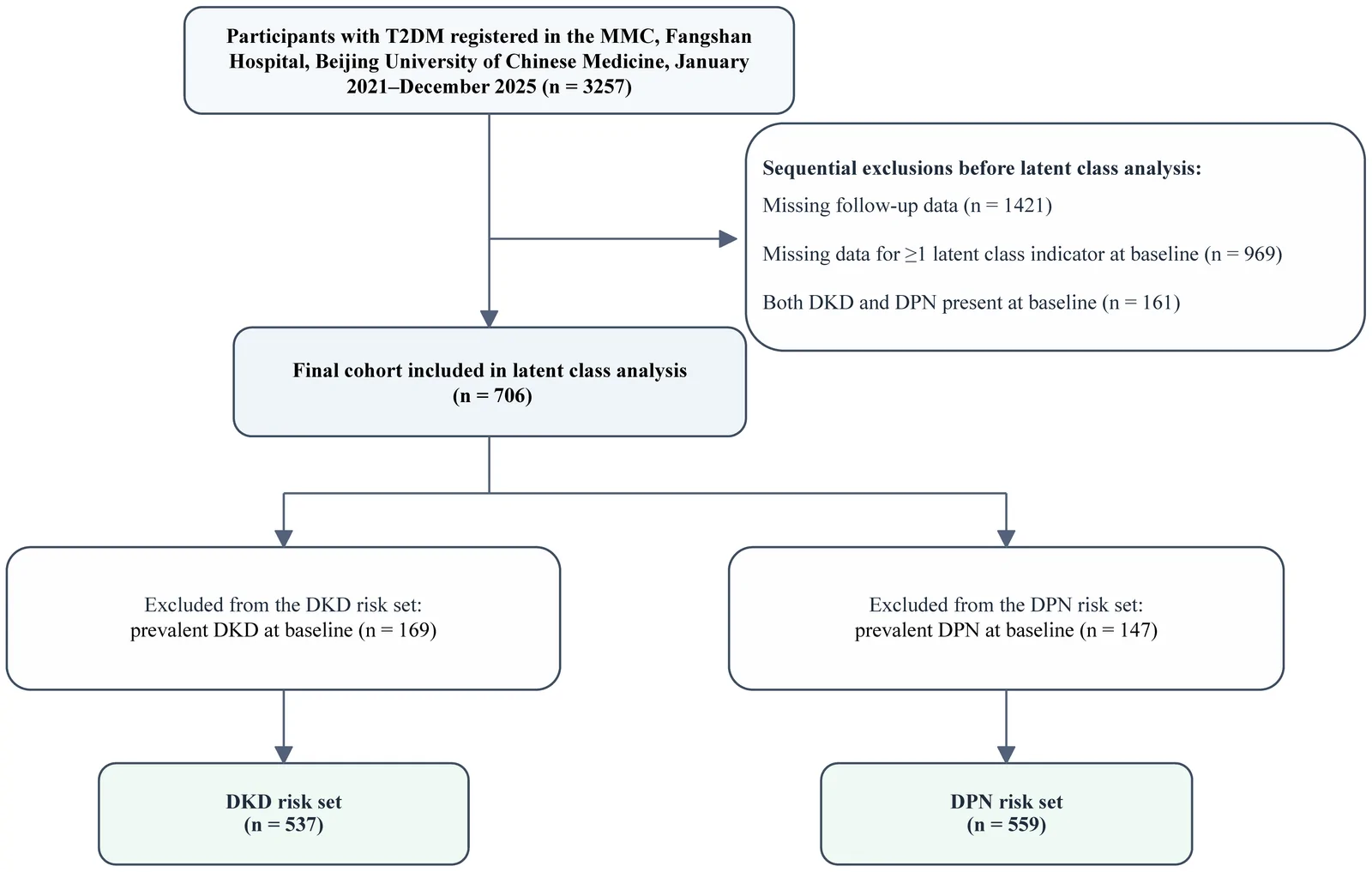
Flowchart of the study.

### 2.2 Variables

Candidate indicators were selected based on clinical relevance and previously published evidence (9, 11, 19–21). Eleven indicators derived from standardized MMC assessments were included in the LCA model, comprising five behavioral factors (smoking status, alcohol consumption, physical activity, sodium intake, and fruit and vegetable intake) and six metabolic factors (blood pressure, body mass index [BMI], visceral fat area [VFA], homeostasis model assessment of insulin resistance based on C-peptide [HOMA-CP], glycated hemoglobin [HbA1c], and triglyceride-to-high-density lipoprotein cholesterol ratio [TG/HDL-C]). HOMA-CP was calculated as 1.5 + [FPG (mmol/L) × FCP (pmol/L)]/2800. This C-peptide-based index was used because some participants were receiving exogenous insulin therapy. Blood pressure control was defined as controlled (SBP <140 mmHg and DBP <90 mmHg) or uncontrolled (SBP ≥140 mmHg or DBP ≥90 mmHg) (22), and glycemic control was classified as controlled (HbA1c <7.0%) or uncontrolled (HbA1c ≥7.0%) (23). All indicators were categorized and entered into the LCA model to identify distinct behavioral-metabolic risk patterns. Detailed classification criteria are provided in **Supplementary Table S1**. Covariates included sociodemographic characteristics, diabetes-related characteristics, and glucose-lowering medication use. SES was constructed as a composite indicator based on education level, occupation, and annual household income (9). A total SES score ranging from 0 to 6 was calculated, with higher scores indicating higher SES. Participants were subsequently categorized into low SES (0-4 points) and high SES (5-6 points) groups (24).

### 2.3 Outcomes

The primary outcomes were incident DKD and incident DPN during follow-up. Incident DKD was defined as newly diagnosed DKD during follow-up, requiring persistent UACR ≥30 mg/g and/or eGFR <60 mL/(min·1.73 m²) for at least 3 months, according to KDIGO recommendations for chronic kidney disease assessment (25). The date when these criteria were first met was considered the incident DKD date for survival analysis. DPN was diagnosed according to the Toronto consensus criteria for typical distal symmetric polyneuropathy (DSPN), with confirmed DSPN defined as abnormal nerve conduction findings accompanied by neuropathic symptoms and/or neurological signs, after exclusion of other potential causes of peripheral neuropathy (26). Nerve conduction was assessed using the DPNCheck device (NeuroMetrix, USA). Baseline nerve conduction data were available for all participants. Follow-up duration was calculated from the baseline visit date to the outcome-specific event or censoring date. Median follow-up was estimated using the reverse Kaplan–Meier method.

### 2.4 Statistical analysis

Baseline characteristics are presented as medians with interquartile ranges (IQRs) for continuous variables and frequencies with percentages for categorical variables. Missing data for all covariates were below 10%. Multiple imputation by chained equations was performed to generate 20 imputed datasets, with 5 iterations per imputation procedure. Multinomial logistic regression and Cox proportional hazards models were fitted separately in each imputed dataset, and estimates were pooled using Rubin’s rules.

To identify behavioral-metabolic phenotypes among individuals with T2DM, latent class analysis (LCA) was performed based on 11 behavioral and metabolic indicators. Models with one to six latent classes were fitted sequentially, and the optimal number of classes was determined based on the Akaike information criterion (AIC), Bayesian information criterion (BIC), adjusted BIC (aBIC), entropy, and clinical interpretability. Based on the highest posterior probability of class membership, participants were assigned to the corresponding latent class (27). Univariable and multivariable logistic regression models were used to examine associations between demographic characteristics, clinical factors, SES, and latent class assignment. Results are presented as odds ratios (ORs) and 95% confidence intervals (CIs). Kaplan–Meier curves were used to visualize event-free survival for incident DKD and DPN across the three behavioral-metabolic phenotypes, and differences between survival curves were assessed using the log-rank test. Cox proportional hazards regression models were used to assess associations between latent classes and the risks of incident DKD and DPN. Results are presented as hazard ratios (HRs) and 95% CIs. Three sequential models were constructed: the unadjusted model included no covariate adjustments; Model 1 was adjusted for demographic characteristics (age and sex); Model 2 further adjusted for diabetes duration, fasting plasma glucose, and glucose-lowering medications, including SGLT2 inhibitors, GLP-1 receptor agonists, and insulin. Additionally, exploratory SES-stratified Cox proportional hazards regression analyses were conducted to examine phenotype–complication associations across different SES strata. Given the limited number of outcome events within individual strata, these analyses were considered exploratory. Because glycemic and blood pressure control were components of the latent phenotype definition, baseline HbA1c, SBP, and DBP were not included in the primary Cox models. Sensitivity analyses were therefore conducted by further adjusting Model 2 for these variables and by repeating the LCA with the TyG index replacing HOMA-CP. An additional exploratory TyG-based analysis excluding TG/HDL-C was performed to reduce overlapping triglyceride-related information. The proportional hazards (PH) assumption was checked using scaled Schoenfeld residuals, and all models satisfied this assumption. All statistical analyses were performed using R software (version 4.4.0), and a two-sided *p* < 0.05 was considered statistically significant.

## 3 Results

### 3.1 Latent class analysis

Based on the model fit indices, the three-class model was selected as the optimal solution. Although the AIC and aBIC continued to decrease in the four-class model, the three-class solution had the lowest BIC and higher entropy. The class-specific average posterior probabilities (AvePP) for the three-class solution were all above 0.90, whereas the four-class solution showed lower assignment certainty for one class (AvePP = 0.806), with partial overlap with another class. Considering model fit, classification certainty, parsimony, and clinical interpretability, the three-class solution was retained (**Tables 1**, **2**; **Supplementary Table S2**).

**Table 1:** Indicators of model fit.

LCA model	1-class	2-class	3-class	4-class	5-class	6-class
LL	-6541.45	-6323.50	-6217.45	-6175.71	-6137.51	-6099.47
AIC	13118.90	12721.00	12546.91	12501.42	12463.01	12424.95
BIC	13200.97	12889.71	12802.25	12843.39	12891.62	12940.19
aBIC	13143.82	12772.23	12624.44	12605.25	12593.15	12581.38
Entropy	1.000	0.744	0.846	0.807	0.744	0.765
Class proportion (%)	N/A	53.1/46.9	14.3/39.8/45.9	12.4/42.0/18.3/27.3	30.4/18.0/15.1/12.5/23.9	31.0/15.1/17.1/8.1/5.4/23.3

Model selection was based on log-likelihood, Akaike information criterion, Bayesian information criterion, adjusted Bayesian information criterion, entropy, and class proportions. aBIC, adjusted Bayesian information criterion; AIC, Akaike information criterion; BIC, Bayesian information criterion; LCA, latent class analysis; LL, log-likelihood.

**Table 2:** Average posterior probabilities for individual assignment in the latent class model.

Final assignment	Class 1	Class 2	Class 3
Class 1	0.907	0.054	0.039
Class 2	0.019	0.942	0.039
Class 3	0.007	0.060	0.933

Class 1 (14.3%) was identified as the Behavioral-Visceral Adiposity (BVA) phenotype, characterized by the clustering of unfavorable lifestyle behaviors, including smoking, alcohol consumption, high sodium intake, insufficient fruit and vegetable intake, and physical inactivity. Notably, this phenotype exhibited relatively low BMI but increased VFA, consistent with the “thin outside, fat inside” (TOFI) profile, accompanied by dyslipidemia and moderate insulin resistance. Class 2 (39.8%) was identified as the Obesity-Related Metabolic Risk (OMR) phenotype, characterized by higher probabilities of obesity, physical inactivity, insulin resistance, hypertension, and an elevated TG/HDL-C ratio. Class 3 (45.9%) was identified as the Low-Risk (LR) phenotype, characterized by relatively favorable behavioral patterns and metabolic profiles (**Figure 2**).

**Figure 2:**
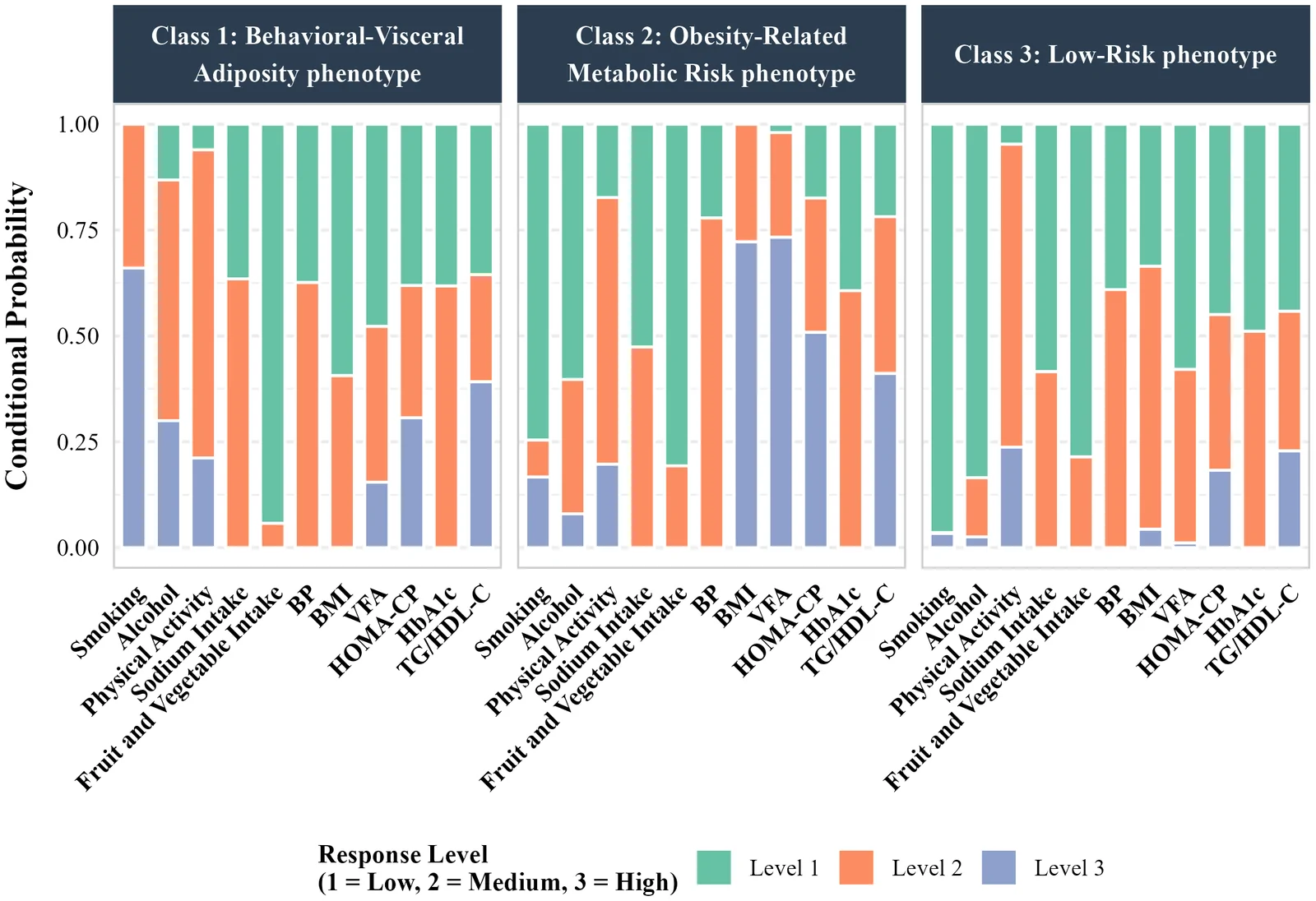
Characteristics and distribution probabilities of latent behavioral-metabolic risk phenotypes in patients with type 2 diabetes mellitus. This figure indicates the identification of three distinct patient phenotypes—Behavioral-Visceral Adiposity phenotype, Obesity-Related Metabolic Risk phenotype, and Low-Risk phenotype—based on their varying response levels (low, medium, or high) to specific behavioral habits and metabolic health markers. BMI, body mass index; BP, blood pressure; HbA1c, glycated hemoglobin; HOMA-CP, homeostasis model assessment of insulin resistance based on C-peptide; LCA, latent class analysis; TG/HDL-C, triglyceride-to-high-density lipoprotein cholesterol ratio; VFA, visceral fat area.

### 3.2 Baseline characteristics

**Table 3** presents the baseline characteristics of participants across the three latent classes. Individuals in the BVA phenotype had the lowest BMI but relatively elevated VFA. This phenotype consisted predominantly of younger men with higher educational attainment, non-manual occupations, and higher household income. Despite these favorable socioeconomic characteristics, this phenotype exhibited the most pronounced clustering of unhealthy behaviors, including higher proportions of smoking, alcohol consumption, insufficient fruit and vegetable intake, and high sodium intake. The OMR phenotype demonstrated the most pronounced metabolic abnormalities, with the highest levels of BMI, VFA, HOMA-CP, and prevalence of hypertension. Individuals in this phenotype also showed the highest prevalence of physical inactivity, representing a profile characterized by the coexistence of obesity, visceral adiposity, and insulin resistance. The LR phenotype included a higher proportion of women and older individuals with longer diabetes duration, but exhibited the most favorable behavioral patterns and metabolic profiles, including lower BMI, VFA, and HOMA-CP levels and healthier lifestyle behaviors.

**Table 3:** Baseline characteristics of participants across the three latent classes.

Variables	Total (n=706)	BVA phenotype (n=101)	OMR phenotype (n=281)	LR phenotype (n=324)	*P*
Demographic characteristics					
Sex, n (%)					<0.001
Female	352 (49.9)	10 (9.9)	130 (46.3)	212 (65.4)	
Male	354 (50.1)	91 (90.1)	151 (53.7)	112 (34.6)	
Age (years), median (IQR)	60.0 (52.3, 68.0)	57.0 (50.0, 62.0)	59.0 (50.0, 68.0)	61.0 (55.0, 69.0)	<0.001
Educational level, n (%)					<0.001
<High school	335 (47.5)	22 (21.8)	143 (50.9)	170 (52.5)	
≥ High school	371 (52.5)	79 (78.2)	138 (49.1)	154 (47.5)	
Occupation, n (%)					0.002
Unemployed	167 (23.7)	11 (10.9)	69 (24.6)	87 (26.9)	
Manual worker	59 (8.4)	4 (4.0)	29 (10.3)	26 (8.0)	
Non-manual worker	480 (68.0)	86 (85.1)	183 (65.1)	211 (65.1)	
Annual family income, n (%)					0.067
<30,000 CNY	27 (3.8)	3 (3.0)	11 (3.9)	13 (4.0)	
30,000-99,999 CNY	248 (35.1)	23 (22.8)	105 (37.4)	120 (37.0)	
≥100,000 CNY	431 (61.0)	75 (74.3)	165 (58.7)	191 (59.0)	
Behavioral characteristics					
Smoking behavior, n (%)					<0.001
Non-smoker	518 (73.4)	0 (0.0)	211 (75.1)	307 (94.8)	
Occasional smoker	61 (8.6)	35 (34.7)	26 (9.3)	0 (0.0)	
Current smoker	127 (18.0)	66 (65.3)	44 (15.7)	17 (5.2)	
Alcohol consumption, n (%)					<0.001
Non-drinker	449 (63.6)	5 (5.0)	171 (60.9)	273 (84.3)	
Occasional drinker	195 (27.6)	63 (62.4)	88 (31.3)	44 (13.6)	
Regular drinker	62 (8.8)	33 (32.7)	22 (7.8)	7 (2.2)	
Physical activity, n (%)					<0.001
Low	72 (10.2)	6 (5.9)	52 (18.5)	14 (4.3)	
Moderate	481 (68.1)	73 (72.3)	175 (62.3)	233 (71.9)	
Vigorous	153 (21.7)	22 (21.8)	54 (19.2)	77 (23.8)	
Sodium intake, n (%)					<0.001
≤2.3 g/day	373 (52.8)	36 (35.6)	149 (53.0)	188 (58.0)	
>2.3 g/day	333 (47.2)	65 (64.4)	132 (47.0)	136 (42.0)	
Fruit and vegetable intake, n (%)					0.006
Inadequate	577 (81.7)	94 (93.1)	226 (80.4)	257 (79.3)	
Adequate	129 (18.3)	7 (6.9)	55 (19.6)	67 (20.7)	
Clinical and metabolic characteristics					
Duration of T2DM (months), median (IQR)	86 (26, 162)	95 (30, 144)	63 (13, 157)	99 (38, 172)	0.014
Family history of T2DM, n (%)					0.469
No	404 (57.2)	53 (52.5)	167 (59.4)	184 (56.8)	
Yes	302 (42.8)	48 (47.5)	114 (40.6)	140 (43.2)	
BMI (kg/m²), median (IQR)	26.3 (24.2, 29.0)	23.8 (23.3, 25.5)	29.5 (28.1, 31.2)	24.9 (23.4, 26.3)	<0.001
VFA (cm²), median (IQR)	111.5 (85.0, 139.0)	103.0 (77.0, 124.0)	145.0 (129.0, 168.0)	89.0 (70.0, 106.3)	<0.001
HbA1c (%), median (IQR)	7.2 (6.3, 8.5)	7.5 (6.6, 8.5)	7.2 (6.5, 8.5)	7.1 (6.3, 8.5)	0.335
HOMA-CP, median (IQR)	3.9 (3.0, 5.1)	3.7 (3.0, 4.9)	4.5 (3.6, 5.8)	3.4 (2.8, 4.3)	<0.001
TG/HDL-C ratio, median (IQR)	1.34 (0.88, 1.99)	1.49 (0.87, 2.60)	1.54 (1.07, 2.23)	1.11 (0.70, 1.64)	<0.001
Blood pressure control, n (%)					<0.001
Controlled	224 (31.7)	35 (34.7)	63 (22.4)	126 (38.9)	
Uncontrolled	482 (68.3)	66 (65.3)	218 (77.6)	198 (61.1)	
Glycemic control, n (%)					0.113
Controlled	306 (43.3)	39 (38.6)	113 (40.2)	154 (47.5)	
Uncontrolled	400 (56.7)	62 (61.4)	168 (59.8)	170 (52.5)	
Medication use, n (%)					
Oral antidiabetic drug use	477 (67.6)	69 (68.3)	176 (62.6)	232 (71.6)	0.102
Insulin therapy	207 (29.3)	31 (30.7)	80 (28.5)	96 (29.6)	0.903
SGLT2 inhibitor use	132 (18.7)	28 (27.7)	46 (16.4)	58 (17.9)	0.038
GLP-1 receptor agonist use	30 (4.2)	4 (4.0)	19 (6.8)	7 (2.2)	0.019

Continuous variables are presented as median (interquartile range [IQR]) and categorical variables are presented as counts (percentages, %). p values were calculated using the Kruskal-Wallis test for continuous variables and the Chi-square test (or Fisher’s exact test) for categorical variables. BMI, body mass index; BVA, Behavioral-Visceral Adiposity; CNY, Chinese Yuan; GLP-1, glucagon-like peptide-1; HbA1c, glycated haemoglobin; HOMA-CP, homeostasis model assessment of insulin resistance based on C-peptide; LR, Low-Risk; OMR, Obesity-Related Metabolic Risk; SGLT2, sodium-glucose cotransporter 2; T2DM, type 2 diabetes mellitus; TG/HDL-C, triglyceride-to-high-density lipoprotein cholesterol ratio; VFA, visceral fat area.

### 3.3 Predictors of behavioral-metabolic latent class membership

Univariable multinomial logistic regression analysis was performed using the LR class as the reference group (**Table 4**). Age and sex were significantly associated with membership in the BVA and OMR classes (both *p* < 0.001). Older age was inversely associated with membership in the BVA class (OR = 0.96, 95% CI = 0.94-0.98) and OMR class (OR = 0.97, 95% CI = 0.96-0.98). Conversely, males had a higher probability of being in the BVA class (OR = 17.18, 95% CI = 8.61-34.30) and OMR class (OR = 2.20, 95% CI = 1.58-3.05) compared to females. In addition, compared to the low risk class, non-manual workers (OR = 3.22, 95% CI = 1.64-6.33, *p* < 0.001) and individuals with higher educational levels (OR = 3.96, 95% CI = 2.36-6.67, *p* < 0.001) were more likely to belong to the BVA class. After adjustment for potential confounders, SES remained independently associated with latent class membership. Higher SES was positively associated with the BVA class membership (OR = 2.04, 95% CI = 1.16-3.57, *p* = 0.013) but inversely associated with the OMR class membership (OR = 0.646, 95% CI = 0.445-0.939, *p* = 0.022).

**Table 4:** Multinomial logistic regression analysis of predictors for latent class membership.

Variables	BVA phenotype: unadjusted OR (95% CI)	*P*	BVA phenotype: adjusted OR (95% CI)†	*P*	OMR phenotype: unadjusted OR (95% CI)	*P*	OMR phenotype: adjusted OR (95% CI)†	*P*
Age (years)	0.96 (0.94-0.98)	<0.001	0.977 (0.954-1.00)	0.064	0.97 (0.96-0.98)	<0.001	0.969 (0.953-0.986)	<0.001
Sex (male)	17.18 (8.61-34.30)	<0.001	14.20 (7.03-28.90)	<0.001	2.20 (1.58-3.05)	<0.001	2.30 (1.63-3.25)	<0.001
Duration of T2DM (months)	0.999 (0.996-1.00)	0.289	1.000 (0.997-1.00)	0.763	0.998 (0.996-1.00)	0.015	0.999 (0.997-1.00)	0.186
Family history of T2DM	1.19 (0.76-1.86)	0.446	1.24 (0.754-2.04)	0.397	0.90 (0.65-1.24)	0.512	0.897 (0.637-1.26)	0.532
Occupation								
Unemployed	Ref.	—	Ref.	—	Ref.	—	Ref.	—
Manual worker	1.22 (0.36-4.14)	0.754	—	—	1.41 (0.76-2.61)	0.278	—	—
Non-manual worker	3.22 (1.64-6.33)	<0.001	—	—	1.09 (0.75-1.59)	0.638	—	—
Educational level (high school or above)	3.96 (2.36-6.67)	<0.001	—	—	1.07 (0.77-1.47)	0.698	—	—
Annual family income								
<30,000 CNY	Ref.	—	Ref.	—	Ref.	—	Ref.	—
30,000-99,999 CNY	0.83 (0.22-3.15)	0.785	—	—	1.03 (0.44-2.41)	0.938	—	—
≥100,000 CNY	1.70 (0.47-6.14)	0.417	—	—	1.02 (0.45-2.34)	0.961	—	—
SES score	4.20 (2.55-6.90)	<0.001	2.04 (1.16-3.57)	0.013	1.07 (0.77-1.48)	0.689	0.646 (0.445-0.939)	0.022

Multinomial logistic regression was used to identify factors associated with latent class membership. †Adjusted for age, sex, family history of T2DM, and SES score. Occupation, educational level, and annual family income were excluded from the adjusted model because they are components of the composite SES score. LR phenotype was used as the reference group. ORs are presented with 95% CIs. BVA, Behavioral-Visceral Adiposity; CI, confidence interval; CNY, Chinese Yuan; LR, Low-Risk; OR, odds ratio; OMR, Obesity-Related Metabolic Risk; Ref., reference; SES, socioeconomic status; T2DM, type 2 diabetes mellitus.

### 3.4 Health outcomes of the different classes

After excluding individuals with the respective pre-existing complications at baseline, the longitudinal analyses included a risk set of 537 participants for incident DKD and 559 participants for incident DPN. The median follow-up duration was 23.0 months for DKD and 21.9 months for DPN, with total follow-up times of 930.6 and 945.1 person-years, respectively. Kaplan–Meier analyses showed significant differences in event-free survival across the three behavioral-metabolic phenotypes for both DKD (log-rank *p* = 0.003) and DPN (log-rank *p* = 0.011) (**Figure 3**). For DKD, the OMR phenotype showed a lower event-free survival probability than the other phenotypes over follow-up. For DPN, the BVA and OMR phenotypes showed lower event-free survival probabilities than the LR phenotype. These patterns were further evaluated using Cox proportional hazards regression. Compared with the LR phenotype, the OMR phenotype was associated with a significantly increased risk of incident DKD in the fully adjusted model (HR = 2.006, 95% CI = 1.324-3.040, *p* = 0.001), whereas no significant association was observed for the BVA phenotype (HR = 1.007, 95% CI = 0.514-1.972, *p* = 0.985). For incident DPN, both the BVA and OMR phenotypes were associated with increased risks. The BVA phenotype was associated with an approximately 2.5-fold higher risk of DPN (HR = 2.467, 95% CI = 1.189-5.121, *p* = 0.015), whereas the OMR phenotype was associated with an approximately twofold higher risk (HR = 2.056, 95% CI = 1.182-3.576, *p* = 0.011) (**Table 5**).

**Figure 3:**
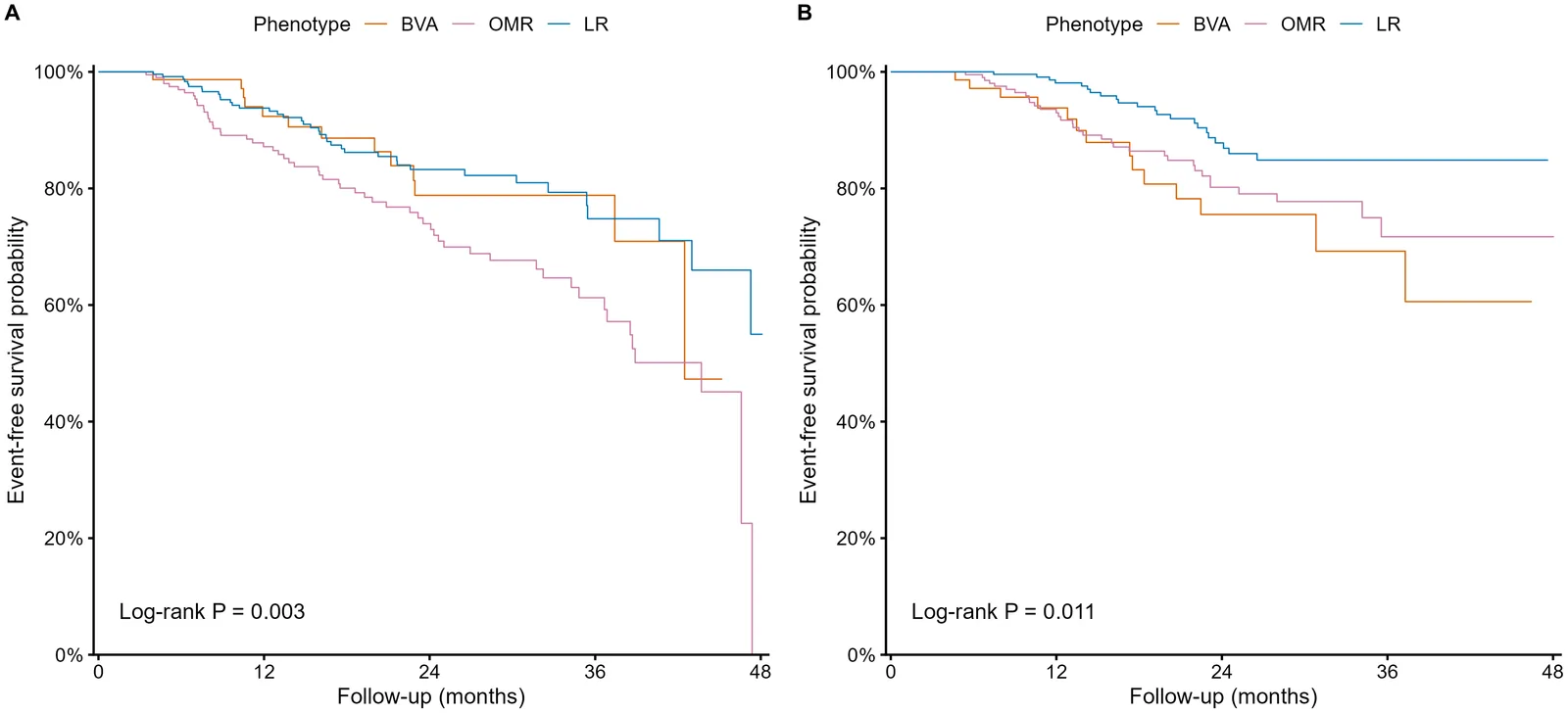
Kaplan–Meier curves for event-free survival according to behavioral-metabolic phenotype. **(A)** DKD-free survival differed across the three behavioral-metabolic phenotypes (log-rank *p* = 0.003). **(B)** DPN-free survival differed across the three phenotypes (log-rank *p* = 0.011). BVA, Behavioral-Visceral Adiposity; DKD, diabetic kidney disease; DPN, diabetic peripheral neuropathy; LR, Low-Risk; OMR, Obesity-Related Metabolic Risk.

**Table 5:** Association of latent behavioral-metabolic phenotypes with risks of incident DKD and DPN.

	Incidents/Population	Unadjusted HR (95% CI)	*P*	Model 1 HR (95% CI)†	*P*	Model 2 HR (95% CI)‡	*P*
DKD							
LR phenotype	39/253	Ref.	—	Ref.	—	Ref.	—
BVA phenotype	13/78	1.128 (0.600-2.120)	0.709	1.145 (0.589-2.226)	0.700	1.007 (0.514-1.972)	0.985
OMR phenotype	58/206	1.968 (1.308-2.962)	0.001	2.015 (1.334-3.044)	<0.001	2.006 (1.324-3.040)	0.001
DPN							
LR phenotype	22/263	Ref.	—	Ref.	—	Ref.	—
BVA phenotype	14/75	2.474 (1.266-4.836)	0.001	2.501 (1.207-5.185)	0.014	2.467 (1.189-5.121)	0.015
OMR phenotype	34/221	1.932 (1.130-3.302)	0.016	2.007 (1.157-3.479)	0.013	2.056 (1.182-3.576)	0.011

Hazard ratios (HRs) with 95% confidence intervals (CIs) were estimated using Cox regression models. †Model 1 adjusted for age and sex. ‡Model 2 additionally adjusted for T2DM duration, fasting plasma glucose, and baseline antidiabetic therapies. BVA, Behavioral-Visceral Adiposity; CI, confidence interval; DKD, diabetic kidney disease; DPN, diabetic peripheral neuropathy; GLP-1, glucagon-like peptide-1; HR, hazard ratio; LR, Low-Risk; OMR, Obesity-Related Metabolic Risk; Ref., reference; SGLT2, sodium-glucose cotransporter 2; T2DM, type 2 diabetes mellitus.

### 3.5 Stratified analyses according to SES

In SES-stratified analyses, the association between the OMR phenotype and DKD risk was primarily observed among individuals with low SES (*p* = 0.040). For DPN, the OMR phenotype was associated with an increased risk among individuals with low SES (*p* = 0.039), whereas the BVA phenotype was associated with an increased risk among individuals with high SES (*p* = 0.046)(**Figure 4**, **Supplementary Table S3**).

**Figure 4:**
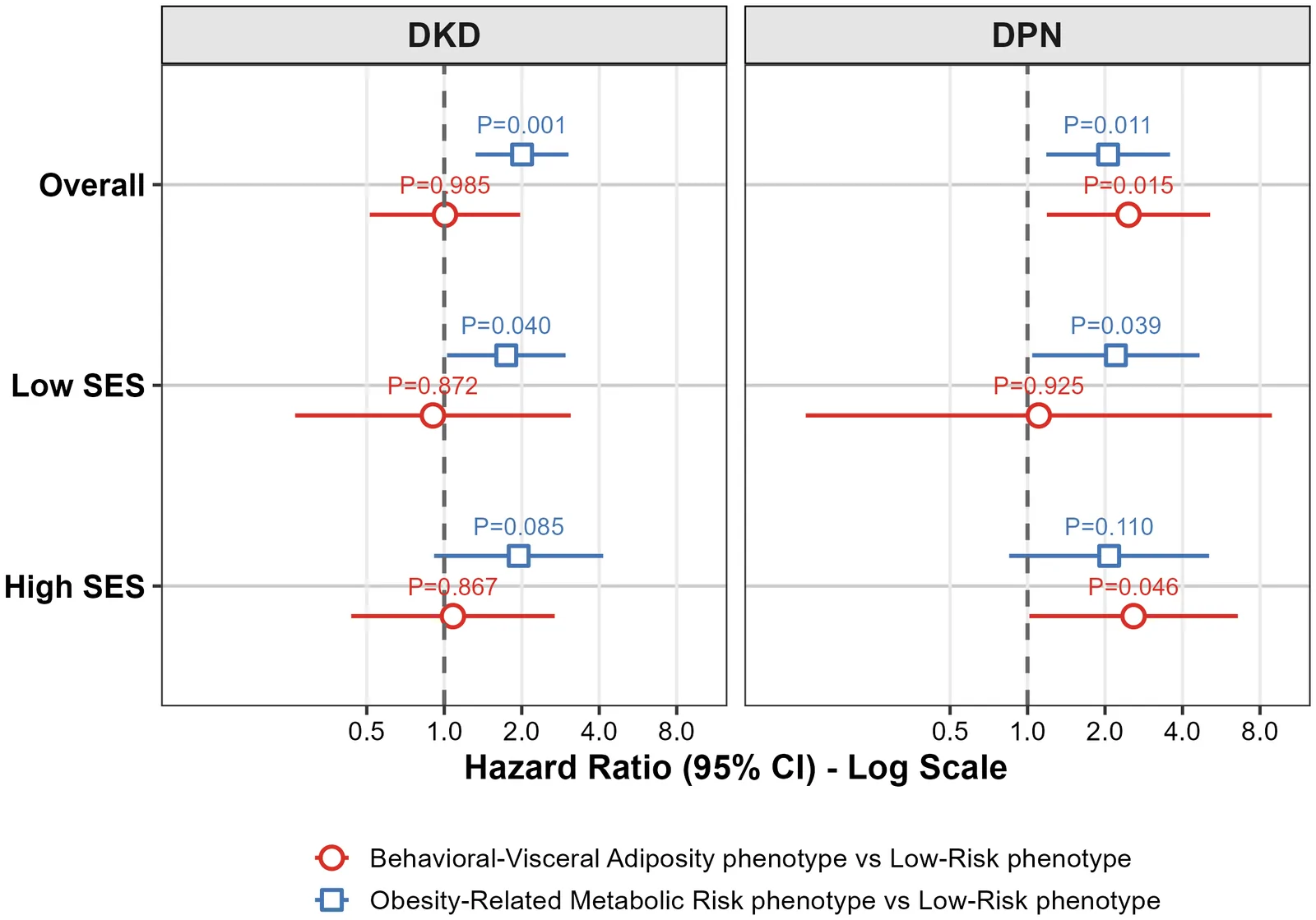
Forest plot of the associations between behavioral-metabolic phenotypes and the risks of diabetic kidney disease and diabetic peripheral neuropathy stratified by socioeconomic status. This figure indicates that, compared with the Low-Risk phenotype, patients in the Obesity-Related Metabolic Risk phenotype have a significantly higher risk of DKD overall and among those with low SES, while the association is attenuated in the high-SES group. For DPN, both the Behavioral-Visceral Adiposity phenotype and the Obesity-Related Metabolic Risk phenotype are associated with a higher overall risk compared with the Low-Risk phenotype; Behavioral-Visceral Adiposity phenotype shows a significant association among individuals with high SES, whereas the Obesity-Related Metabolic Risk phenotype shows a significant association among those with low SES. CI, confidence interval; DKD, diabetic kidney disease; DPN, diabetic peripheral neuropathy; SES, socioeconomic status.

### 3.6 Sensitivity analyses

Further adjustment for baseline HbA1c, SBP, and DBP did not materially alter the results, with the associations of the OMR phenotype with DKD and of the BVA and OMR phenotypes with DPN remaining significant (**Supplementary Table S4**). Replacing HOMA-CP with TyG yielded an acceptable three-class solution (entropy = 0.821), but concordance with the primary classification was modest (agreement = 56.7%; ARI = 0.259), and BIC favored five classes. Because both TyG and TG/HDL-C contain triglyceride-related information, TG/HDL-C was excluded in an additional exploratory analysis. The resulting three-class model had the lowest BIC (11,289.53), good classification quality (entropy = 0.849), and high concordance with the primary classification (agreement = 96.6%; ARI = 0.900) (**Supplementary Tables S5–S7**). Cox analyses based on this TyG-derived classification showed similar risk patterns (**Supplementary Table S8**).

## 4 Discussion

In this study, LCA integrating behavioral and metabolic characteristics identified three distinct behavioral-metabolic phenotypes among individuals with T2DM. These phenotypes showed differential associations with diabetic microvascular complications. Compared with the LR phenotype, the OMR phenotype was associated with increased risks of both incident DKD and DPN, whereas the BVA phenotype was primarily associated with increased DPN risk. Furthermore, SES was independently associated with behavioral-metabolic phenotype membership, and the associations between specific phenotypes and microvascular complications varied across SES strata. Sensitivity analyses further supported the robustness of the primary findings. The observed associations remained consistent after additional adjustment for baseline HbA1c and blood pressure, and the core three-class phenotype structure and complication-risk patterns were reproduced in the TyG-based analysis after minimizing overlapping triglyceride-related information. These findings highlight the potential value of integrating behavioral, metabolic, and social determinants to identify heterogeneous risk profiles among individuals with T2DM and provide additional evidence supporting precision risk stratification for diabetic complications.

Our analysis revealed that individuals in the OMR phenotype had significantly increased risks of both DKD and DPN. These findings are consistent with previous studies indicating that obesity and components of metabolic syndrome are important contributors to diabetic microvascular complications (28–30). Additionally, physical inactivity, as a behavioral contributor to obesity and insulin resistance, has been consistently associated with increased risks of diabetic microvascular complications (31). Sipola G et al. reported that obesity and metabolic syndrome may contribute to neuropathic damage independently of hyperglycemia and may serve as important predictors of diabetic neuropathy onset (32). In this study, individuals in this phenotype exhibited marked obesity, visceral adiposity, and insulin resistance, accompanied by suboptimal blood pressure control and insufficient physical activity, suggesting that behavioral factors and metabolic abnormalities may interact to contribute to microvascular complication risk. Previous studies have suggested that visceral adiposity may contribute to renal and peripheral nerve damage through mechanisms including chronic low-grade inflammation, lipotoxicity, and hemodynamic abnormalities (12, 33, 34). Therefore, the clustering of multiple behavioral and metabolic risk factors may partly explain the increased risk of complications observed in this phenotype.

In contrast, the BVA phenotype exhibited a distinct risk pattern. Individuals in this phenotype had relatively low BMI but increased visceral adiposity, representing a profile consistent with normal-weight obesity (NWO) or the TOFI phenotype. Accumulating evidence indicates that BMI alone may underestimate metabolic risk because individuals with normal BMI may still exhibit substantial visceral adiposity and metabolic dysfunction (35–37). A prospective cohort study among Chinese adults demonstrated that NWO was independently associated with incident T2DM (38). Similarly, Zhong et al. reported that central obesity among individuals with normal BMI was associated with increased risks of diabetic microvascular complications and cardiovascular events (39). These findings highlight the importance of assessing body composition beyond BMI alone for identifying individuals with clinically relevant metabolic abnormalities. Notably, the BVA phenotype identified in our study was characterized not only by high VFA but also by the coexistence of multiple unhealthy lifestyle behaviors. These behavioral factors may contribute to oxidative stress, chronic inflammation, and lipotoxicity, potentially accelerating peripheral nerve injury and contributing to the increased risk of DPN (40). These findings suggest that conventional BMI-based obesity assessment may fail to capture clinically meaningful metabolic risk in certain patients. Therefore, individuals with normal BMI but elevated visceral adiposity and unhealthy lifestyle profiles may benefit from targeted interventions, including visceral adiposity assessment, lifestyle modification, and early DPN screening. The absence of a significant association between the BVA phenotype and DKD, despite its association with DPN, may reflect differences in the underlying pathophysiological mechanisms of diabetic microvascular complications (41). DKD is primarily influenced by renal hemodynamic abnormalities, glomerular injury, and fibrosis pathways (42), whereas DPN involves mechanisms including oxidative stress, mitochondrial dysfunction, lipotoxicity, and Schwann cell injury (43). Therefore, behavioral and metabolic risk factors may have differential effects across target organs. Future studies incorporating mechanistic biomarkers and longer follow-up periods are warranted to further elucidate these potential pathways.

SES represents an important social determinant influencing diabetes management, lifestyle behaviors, healthcare access, and long-term outcomes (17). In the present study, SES was independently associated with behavioral-metabolic phenotype membership. Higher SES was associated with greater likelihood of belonging to the BVA phenotype, whereas lower SES was associated with the OMR phenotype. These findings suggest that socioeconomic conditions may influence the distribution and clustering of behavioral and metabolic risk factors. Several potential explanations may account for these observations. Individuals with higher SES may have greater access to healthcare resources and health information but may also experience occupational sedentary behavior, psychosocial stress, and social drinking patterns, potentially contributing to latent metabolic abnormalities. Conversely, individuals with lower SES may be more vulnerable to unhealthy dietary patterns, limited opportunities for physical activity, and barriers to effective chronic disease management, which may contribute to obesity-related metabolic risk profiles (44–46). Thus, SES may influence not only individual risk factors but also the clustering of behavioral-metabolic phenotypes. SES-stratified analyses further suggested that phenotype-associated risks may differ across socioeconomic contexts. The associations between the OMR phenotype and DKD or DPN were primarily observed among individuals with low SES, whereas the association between the BVA phenotype and DPN was observed among individuals with high SES. However, given the limited number of outcome events within individual strata, these analyses were exploratory and larger prospective studies are needed to confirm these findings.

The findings of this study have several potential clinical implications. Current diabetes management strategies primarily emphasize the control of glycaemia, blood pressure, and lipid levels. However, our findings suggest that combinations of behavioral and metabolic characteristics may provide additional information beyond traditional risk factors (47). Individuals with the OMR phenotype may benefit from interventions targeting weight reduction, increased physical activity, and improvement of insulin resistance. For individuals with the BVA phenotype, normal BMI should not be considered equivalent to low metabolic risk; assessment of visceral adiposity, lifestyle modification, and early DPN screening may be particularly important. Moreover, incorporating SES into risk assessment may facilitate targeted health education and resource allocation, thereby supporting individualized prevention strategies.

This study has several strengths. First, LCA enabled the identification of multidimensional behavioral-metabolic phenotypes rather than relying on isolated risk factors, providing a more comprehensive assessment of heterogeneity among individuals with T2DM. Second, the associations of these phenotypes with two major microvascular complications, DKD and DPN, were evaluated separately, revealing potentially distinct risk patterns. Third, integrating SES as both a determinant of phenotype membership and a stratification factor provided additional insights into the role of social determinants in behavioral-metabolic risk profiles and their associations with diabetic complications.

Several limitations should also be acknowledged. First, this was a single-center retrospective cohort study, and the generalizability of the findings may be limited. Second, the limited sample size and number of outcome events may have reduced statistical power, particularly in the SES-stratified analyses, and may have resulted in less precise effect estimates. Future multicenter prospective studies are warranted to confirm the robustness of these associations. Third, some continuous variables were categorized for LCA modelling, which may have resulted in information loss and reduced discrimination between phenotypes. In addition, several behavioral variables were self-reported and may have been subject to recall bias or misclassification. Fourth, standardized fundus photography was not systematically available for all participants in this retrospective cohort; therefore, diabetic retinopathy could not be reliably evaluated as an additional microvascular outcome. Finally, the relatively short follow-up period may have limited our ability to fully capture progressive microvascular complications. Longer follow-up studies are needed to evaluate long-term associations and potential dose-response relationships.

## 5 Conclusions

Behavioral-metabolic phenotypes identified by LCA were associated with differential risks of DKD and DPN among individuals with T2DM. The OMR phenotype was associated with increased risks of both complications, whereas the BVA phenotype showed a stronger association with DPN. SES was associated with behavioral-metabolic phenotype membership, and SES-stratified analyses further suggested that the associations between behavioral-metabolic phenotypes and microvascular complication risks may vary across socioeconomic contexts; however, further studies are needed to validate these findings. These findings suggest that integrating behavioral characteristics, metabolic profiles, and socioeconomic context into conventional diabetes management may enhance risk stratification and support individualized prevention strategies for diabetic microvascular complications in individuals with T2DM.

## Data Availability

The datasets presented in this article are not readily available because, although the clinical data were de-identified, public sharing is restricted by participant privacy and institutional data protection requirements. The datasets are available from the corresponding author upon reasonable request, subject to appropriate institutional and ethical approval. Requests to access the datasets should be directed to Luying Sun, luyingsun@outlook.com.
