# Supplementary material for: Behavioral-metabolic phenotypes and differential risks of diabetic kidney disease and peripheral neuropathy in type 2 diabetes: a latent class analysis
Source: Front Endocrinol (Lausanne). 2026 Oct 15;17:1962966. doi: 10.3389/fendo.2026.1962966 (PMC13716556; doi:10.3389/fendo.2026.1962966)
Supplement: Supplementary file 1 [file Table1.docx]

Supplementary Material

**Supplementary Table S1. Variables list.**

| **Variables** | **Categorization** |
| --- | --- |
| Behavioral risk factors |  |
| Smoking behavior | 0 = Non-smoker; 1 = Occasional smoker (<1 cigarette/day or <7 cigarettes/week); 2 = Current smoker (daily or almost daily)[1] |
| Alcohol | 0 = Non-drinker; 1 = Occasional drinker (<1 time/week); 2 = Regular drinker (weekly or almost weekly)[2] |
| Physical activity | 0 = Low (<3.0 METs); 1 = Moderate (3.0–5.9 METs); 2 = Vigorous (≥6.0 METs)[3,4] |
| Sodium intake | 0 = ≤2.3 g/day; 1 = >2.3 g/day[5] |
| Fruit and vegetable intake | 0 = Inadequate (<400 g vegetables/day or <200 g fruit/day); 1 = Adequate (≥400 g vegetables/day and ≥200 g fruit/day)[6] |
| Metabolic risk factors |  |
| Blood pressure control | 0 = Controlled (SBP <140 mmHg and DBP <90 mmHg); 1 = Uncontrolled (SBP ≥140 mmHg or DBP ≥90 mmHg)[7] |
| BMI | 0 = Normal Weight (<24.0 kg/m²); 1 = Overweight (24.0–27.9 kg/m²); 2 = Obesity (≥28.0 kg/m²)[8] |
| VFA | 0 = Low (T1); 1 = Moderate (T2); 2 = High (T3) |
| HOMA-CP | 0 = Low (T1); 1 = Moderate (T2); 2 = High (T3) |
| Glycemic control | 0 = Controlled (HbA1c <7.0%); 1 = Uncontrolled (HbA1c ≥7.0%)[9] |
| TG/HDL-C ratio | 0 = Low (T1); 1 = Moderate (T2); 2 = High (T3) |
| Covariates |  |
| Sex | 0 = Female; 1 = Male |
| Educational level | 0 = Below high school; 2 = High school or above |
| Income level | 0 = <30,000 CNY; 1 = 30,000–99,999 CNY; 2 = ≥100,000 CNY |
| Occupation | 0 = Unemployed; 1 = Manual worker; 2 = Non-manual worker |
| Family history of T2DM | 0 = No; 1 = Yes |
| Oral antidiabetic drug use | 0 = No; 1 = Yes |
| SGLT2 inhibitor use | 0 = No; 1 = Yes |
| GLP-1 receptor agonist use | 0 = No; 1 = Yes |
| Insulin therapy | 0 = No; 1 = Yes |

Behavioral and metabolic risk factors were categorized according to predefined clinical criteria. BMI, body mass index; VFA, visceral fat area; HOMA-CP, homeostasis model assessment of insulin resistance based on C-peptide; HbA1c, glycated hemoglobin; TG/HDL-C, triglyceride-to-high-density lipoprotein cholesterol ratio; MET, metabolic equivalent of task; SBP, systolic blood pressure; DBP, diastolic blood pressure; CNY, Chinese Yuan; T2DM, type 2 diabetes mellitus; SGLT2, sodium-glucose cotransporter 2; GLP-1, glucagon-like peptide-1.

**Supplementary Table S2. Average posterior probabilities for the four-class LCA.**

| **Final assignment** | **Class 1** | **Class 2** | **Class 3** | **Class 4** |
| --- | --- | --- | --- | --- |
| Class 1 | 0.914314695314642 | 0.0528538781018056 | 0.0326200862493745 | 0.000211340334177667 |
| Class 2 | 0.00504645916701242 | 0.933780103068037 | 0.0155860515164118 | 0.045587386248539 |
| Class 3 | 0.050452605944262 | 0.0291723250353209 | 0.806028058584779 | 0.114347010435638 |
| Class 4 | 1.40979019579624e-12 | 0.0221034405380389 | 0.0877276578394968 | 0.890168901621055 |

Rows indicate assigned classes and columns indicate mean posterior probabilities of class membership; diagonal values represent class-specific average posterior probabilities. LCA, latent class analysis.

**Supplementary Table S3. Socioeconomic status-stratified associations between latent classes and the risks of diabetic kidney disease and diabetic peripheral neuropathy.**

| **Outcomes / Latent phenotypes** | **Low SES HR (95% CI)** | ***P*** | **High SES HR (95% CI)** | ***P*** |
| --- | --- | --- | --- | --- |
| DKD |  |  |  |  |
| LR phenotype | Ref. | — | Ref. | — |
| BVA phenotype | 0.90 (0.26–3.10) | 0.872 | 1.08 (0.43–2.69) | 0.867 |
| OMR phenotype | 1.74 (1.03–2.96) | 0.040 | 1.95 (0.91–4.14) | 0.085 |
| DPN |  |  |  |  |
| LR phenotype | Ref. | — | Ref. | — |
| BVA phenotype | 1.11 (0.14–8.90) | 0.925 | 2.58 (1.02–6.56) | 0.046 |
| OMR phenotype | 2.20 (1.04–4.66) | 0.039 | 2.08 (0.85–5.08) | 0.110 |

HRs with 95% CIs were estimated using SES-stratified Cox proportional hazards models adjusted for age, sex, T2DM duration, fasting plasma glucose, and baseline glucose-lowering therapies. The LR phenotype was the reference group. BVA, Behavioral-Visceral Adiposity; CI, confidence interval; DKD, diabetic kidney disease; DPN, diabetic peripheral neuropathy; HR, hazard ratio; LR, Low-Risk; OMR, Obesity-Related Metabolic Risk; SES, socioeconomic status; T2DM, type 2 diabetes mellitus.

**Supplementary Table S4. Sensitivity analysis of latent phenotype associations with incident DKD and DPN after additional adjustment for baseline HbA1c and blood pressure.**

| **Outcomes / Latent phenotypes** | **Incidents / Population** | **Model 2 HR (95% CI)†** | ***P*** | **Sensitivity model HR (95% CI)‡** | ***P*** |
| --- | --- | --- | --- | --- | --- |
| DKD |  |  |  |  |  |
| LR phenotype | 39 / 253 | Ref. | — | Ref. | — |
| BVA phenotype | 13 / 78 | 1.007 (0.514-1.972) | 0.985 | 1.046 (0.530-2.064) | 0.897 |
| OMR phenotype | 58 / 206 | 2.006 (1.324-3.040) | 0.001 | 2.200 (1.427-3.393) | <0.001 |
| DPN |  |  |  |  |  |
| LR phenotype | 22 / 263 | Ref. | — | Ref. | — |
| BVA phenotype | 14 / 75 | 2.467 (1.189-5.121) | 0.015 | 2.583 (1.236-5.398) | 0.012 |
| OMR phenotype | 34 / 221 | 2.056 (1.182-3.576) | 0.011 | 2.273 (1.283-4.026) | 0.005 |

HRs with 95% CIs were estimated using Cox proportional hazards models. †Model 2 was adjusted for age, sex, T2DM duration, fasting plasma glucose, and baseline use of SGLT2 inhibitors, GLP-1 receptor agonists, and insulin. ‡The sensitivity model was further adjusted for baseline HbA1c, SBP, and DBP. The LR phenotype was the reference group. BVA, Behavioral-Visceral Adiposity; CI, confidence interval; DBP, diastolic blood pressure; DKD, diabetic kidney disease; DPN, diabetic peripheral neuropathy; HbA1c, glycated hemoglobin; HR, hazard ratio; LR, Low-Risk; OMR, Obesity-Related Metabolic Risk; SBP, systolic blood pressure; T2DM, type 2 diabetes mellitus.

**Supplementary Table S5. Model fit indices for the TyG-based LCA with TG/HDL-C retained.**

| **LCA model** | **1-class** | **2-class** | **3-class** | **4-class** | **5-class** | **6-class** |
| --- | --- | --- | --- | --- | --- | --- |
| **LL** | -6541.57 | -6278.78 | -6163.96 | -6095.93 | -6023.21 | -5973.11 |
| **AIC** | 13119.14 | 12631.56 | 12439.93 | 12341.86 | 12234.42 | 12172.22 |
| **BIC** | 13201.22 | 12800.27 | 12695.27 | 12683.83 | **12663.03** | 12687.46 |
| **aBIC** | 13144.06 | 12682.78 | 12517.46 | 12445.69 | **12364.56** | 12328.66 |
| **Entropy** | 1.000 | 0.814 | 0.821 | 0.812 | 0.830 | 0.852 |
| **Class proportion (%)** | N/A | 44.2 / 55.8 | 25.1 / 36.3 / 38.6 | 22.4 / 34.2 / 16.2 / 27.2 | 20.1 / 21.0 / 12.1 / 24.4 / 22.4 | 23.3 / 7.9 / 20.8 / 21.7 / 10.2 / 16.1 |

HOMA-CP was replaced with TyG, while all other LCA indicators, including TG/HDL-C, were retained. aBIC, adjusted Bayesian information criterion; AIC, Akaike information criterion; BIC, Bayesian information criterion; HOMA-CP, homeostasis model assessment of insulin resistance based on C-peptide; LCA, latent class analysis; LL, log-likelihood; TG/HDL-C, triglyceride-to-high-density lipoprotein cholesterol ratio; TyG, triglyceride-glucose index.

**Supplementary Table S6. Model fit indices for the TyG-based LCA after excluding TG/HDL-C.**

| **LCA model** | **1-class** | **2-class** | **3-class** | **4-class** | **5-class** | **6-class** |
| --- | --- | --- | --- | --- | --- | --- |
| **LL** | -5765.99 | -5587.41 | -5480.77 | -5444.67 | -5411.37 | -5377.71 |
| **AIC** | 11563.98 | 11240.83 | 11061.55 | 11023.34 | 10990.73 | 10957.41 |
| **BIC** | 11636.93 | 11391.29 | **11289.53** | 11328.83 | 11373.74 | 11417.94 |
| **aBIC** | 11586.13 | 11286.51 | **11130.77** | 11116.09 | 11107.02 | 11097.24 |
| **Entropy** | 1.000 | 0.757 | 0.849 | 0.797 | 0.813 | 0.813 |
| **Class proportion (%)** | N/A | 44.8 / 55.2 | 14.9 / 39.9 / 45.2 | 24.6 / 44.0 / 13.0 / 18.4 | 24.8 / 44.0 / 16.7 / 4.8 / 9.6 | 9.1 / 37.3 / 5.9 / 19.9 / 9.4 / 18.3 |

HOMA-CP was replaced with TyG, and TG/HDL-C was excluded to reduce overlapping triglyceride-related information. aBIC, adjusted Bayesian information criterion; AIC, Akaike information criterion; BIC, Bayesian information criterion; HOMA-CP, homeostasis model assessment of insulin resistance based on C-peptide; LCA, latent class analysis; LL, log-likelihood; TG/HDL-C, triglyceride-to-high-density lipoprotein cholesterol ratio; TyG, triglyceride-glucose index.

**Supplementary Table S7. Classification performance and concordance between HOMA-CP-based and TyG-based three-class LCA.**

| **Analysis** | **Entropy** | **AvePP** | **Agreement (%)** | **ARI** | **Class retention, n/N (%)** |
| --- | --- | --- | --- | --- | --- |
| HOMA-CP-based LCA | 0.846 | 0.907 / 0.942 / 0.933 | Ref. | Ref. | — |
| TyG-based LCA (TG/HDL-C retained) | 0.821 | **0.956 / 0.911 / 0.896** | 56.7 | 0.259 | **Class 1: 44/101 (43.6%); Class 2: 140/281 (49.8%); Class 3: 216/324 (66.7%)** |
| TyG-based LCA (TG/HDL-C excluded) | **0.849** | **0.900 / 0.944 / 0.937** | **96.6** | **0.900** | Class 1: 99/101 (98.0%); Class 2: 268/281 (95.4%); Class 3: 315/324 (97.2%) |

Overall agreement and ARI were calculated after aligning the TyG-derived class labels with the corresponding primary classes according to their conditional probability profiles. Class retention represents the number and percentage of participants from each primary class who remained in the corresponding class in the TyG-based solution. ARI, adjusted Rand index; AvePP, average posterior probability; HOMA-CP, homeostasis model assessment of insulin resistance based on C-peptide; TG/HDL-C, triglyceride-to-high-density lipoprotein cholesterol ratio; TyG, triglyceride-glucose index.

**Supplementary Table S8. Associations of HOMA-CP-based and TyG-based latent phenotypes with incident DKD and DPN.**

| **Outcomes / Latent phenotypes** | **HOMA-CP-based model HR (95% CI)†** | ***P*** | **TyG-based model HR(95% CI)‡** | ***P*** |
| --- | --- | --- | --- | --- |
| DKD |  |  |  |  |
| LR phenotype | Ref. | — | Ref. | — |
| BVA phenotype | 1.007 (0.514–1.972) | 0.985 | 1.031 (0.531–2.002) | 0.928 |
| OMR phenotype | 2.006 (1.324–3.040) | 0.001 | 2.193 (1.439–3.343) | <0.001 |
| DPN |  |  |  |  |
| LR phenotype | Ref. | — | Ref. | — |
| BVA phenotype | 2.467 (1.189–5.121) | 0.015 | 2.335 (1.144–4.767) | 0.020 |
| OMR phenotype | 2.056 (1.182–3.576) | 0.011 | 1.929 (1.111–3.350) | 0.020 |

HRs with 95% CIs were estimated using Cox proportional hazards models. †The primary LCA included HOMA-CP and TG/HDL-C. ‡The TyG-based LCA replaced HOMA-CP with TyG and excluded TG/HDL-C. Both Cox models were adjusted for age, sex, T2DM duration, fasting plasma glucose, and baseline use of SGLT2 inhibitors, GLP-1 receptor agonists, and insulin. The LR phenotype was the reference group. BVA, Behavioral-Visceral Adiposity; CI, confidence interval; DKD, diabetic kidney disease; DPN, diabetic peripheral neuropathy; HR, hazard ratio; LR, Low-Risk; OMR, Obesity-Related Metabolic Risk; T2DM, type 2 diabetes mellitus; TG/HDL-C, triglyceride-to-high-density lipoprotein cholesterol ratio; TyG, triglyceride-glucose index.

**References**

1.	Inoue-Choi M, Christensen CH, Rostron BL, Cosgrove CM, Reyes-Guzman C, Apelberg B, et al. Dose-Response Association of Low-Intensity and Nondaily Smoking With Mortality in the United States. JAMA Netw Open (2020) 3:e206436. doi: 10.1001/jamanetworkopen.2020.6436

2.	Ye X-F, Miao C-Y, Zhang W, Ji L-N, Wang J-G, ATTEND investigators. Alcohol intake and dyslipidemia in male patients with hypertension and diabetes enrolled in a China multicenter registry. J Clin Hypertens (Greenwich) (2023) 25:183–190. doi: 10.1111/jch.14638

3.	Craig CL, Marshall AL, Sjöström M, Bauman AE, Booth ML, Ainsworth BE, et al. International physical activity questionnaire: 12-country reliability and validity. Med Sci Sports Exerc (2003) 35:1381–1395. doi: 10.1249/01.MSS.0000078924.61453.FB

4.	Strath SJ, Kaminsky LA, Ainsworth BE, Ekelund U, Freedson PS, Gary RA, et al. Guide to the assessment of physical activity: Clinical and research applications: a scientific statement from the American Heart Association. Circulation (2013) 128:2259–2279. doi: 10.1161/01.cir.0000435708.67487.da

5.	American Diabetes Association Professional Practice Committee for Diabetes*. 5. Facilitating Positive Health Behaviors and Well-being to Improve Health Outcomes: Standards of Care in Diabetes-2026. Diabetes Care (2026) 49:S89–S131. doi: 10.2337/dc26-S005

6.	Diet, nutrition and the prevention of chronic diseases: report of a joint WHO/FAO expert consultation, Geneva, 28 January - 1 February 2002. https://www.who.int/publications/i/item/924120916X [Accessed July 6, 2026]

7.	Liu J. Highlights of the 2018 Chinese hypertension guidelines. Clin Hypertens (2020) 26:8. doi: 10.1186/s40885-020-00141-3

8.	Zhou B-F, Cooperative Meta-Analysis Group of the Working Group on Obesity in China. Predictive values of body mass index and waist circumference for risk factors of certain related diseases in Chinese adults--study on optimal cut-off points of body mass index and waist circumference in Chinese adults. Biomed Environ Sci (2002) 15:83–96.

9.	ElSayed NA, Aleppo G, Aroda VR, Bannuru RR, Brown FM, Bruemmer D, et al. 6. Glycemic Targets: Standards of Care in Diabetes-2023. Diabetes Care (2023) 46:S97–S110. doi: 10.2337/dc23-S006
